# *miR-196b-5p*-mediated downregulation of FAS promotes NSCLC progression by activating IL6-STAT3 signaling

**DOI:** 10.1038/s41419-020-02997-7

**Published:** 2020-09-22

**Authors:** Xiangjie Huang, Sisi Xiao, Xinping Zhu, Yun Yu, Meng Cao, Xiaodong Zhang, Shaotang Li, Wangyu Zhu, Fengjiao Wu, Xiaohui Zheng, Libo Jin, Congying Xie, Xiaoying Huang, Peng Zou, Xiaokun Li, Ri Cui

**Affiliations:** 1 Cancer and Anticancer Drug Research Center, School of Pharmaceutical Sciences, Wenzhou Medical University, Wenzhou, Zhejiang 325035 China; 2 Affiliated Hospital 1, Wenzhou Medical University, Wenzhou, Zhejiang 325035 China; 3 Affiliated Zhoushan Hospital, School of Pharmaceutical Sciences, Wenzhou Medical University, Wenzhou, Zhejiang 325035 China; 4 Institute of Life Sciences, Wenzhou University, Wenzhou, Zhejiang 325035 China; 5 Wenzhou University-Wenzhou Medical University Collaborative Innovation Center of Biomedical, Wenzhou, Zhejiang 325035 China

**Keywords:** Non-small-cell lung cancer, miRNAs

## Abstract

Our recent study demonstrated that the QKI-5 regulated miRNA, *miR-196b-5p*, and it functions as an onco-microRNA in non-small cell lung cancer (NSCLC) by directly targeting GATA6 and TSPAN12. However, the role of *miR-196b-5p* in NSCLC progression and metastasis still remains unclear. We found that *miR-196b-5p* promotes lung cancer cell proliferation and colony formation by directly targeting tumor suppressor, FAS. The expression of FAS was significantly downregulated in NSCLC tissue samples and was negatively correlated with the *miR-196b-5p* expression. Knocking down FAS activates NFkB signaling and subsequent IL6 secretion, resulting in phosphorylation of signal transducer and activator of transcription 3 (STAT3) to promote lung cancer cell growth. Our findings indicated that *miR-196b-5p* might exhibit novel oncogenic function by FAS-mediated STAT3 activation in NSCLC, and suggested that targeting the *miR-196b-5p*/FAS/NFkB/IL6/STAT3 pathway might be a promising therapeutic strategy in treating NSCLC.

## Introduction

Lung cancer is the most commonly diagnosed cancer and the leading cause of cancer-related death worldwide, with ~2.1 million new lung cancer cases and 1.8 million deaths were reported in 2018^1^. Non-small cell lung cancer (NSCLC) accounts for ~80–85% of all lung cancer cases. The main histological subtypes of NSCLC are lung adenocarcinoma (ADC, ~40–50% of all lung cancer cases) and squamous cell carcinoma (SCC, ~20–30% of all lung cancer cases)^2^. Several targeted therapies were available for patients with NSCLC; however, most of them are only effective in treating cancer patients with certain genetic backgrounds. In addition, the 5-year relative survival rate of NSCLC is only about 20% due to the late onset of clinical symptoms and inadequate screening methods^3^. Hence, deeply understanding the molecular mechanisms underlying NSCLC tumorigenesis and target molecules for developing novel therapeutic strategies are urgently needed.

microRNAs (miRNAs), a class of small noncoding RNAs, play a key role in cancer pathogenesis by targeting specific mRNAs^4,5^. miRNA negatively regulates mRNA stability and/or repress mRNA translation by targeting the complementary sequences in the 3′-untranslated region (UTR) of target gene^6^. Most of miRNAs function as a tumor suppressor or oncogene in a tissue-specific manner^5^. Accumulating evidence indicated that the dysregulation of miRNAs was closely associated with NSCLC progression and metastasis^7^. For instance, miR-34 inhibits tumor immune evasion to suppress NSCLC progression by targeting PD-L1^8^. miR-548a-3p has been reported to regulate the Warburg effect and NSCLC growth by inhibiting transcription factor SIX1^9^. let-7, a tumor suppressor in breast cancer, reduces breast tumor-initiating cells and inhibits tumor formation in NOD/SCID mice by silencing multiple targets^10^. In addition, let-7 regulates the Warburg effect and tumor progression by directly targeting PDK1^11^. Clinical trials using chemically modified antisense oligonucleotides and/or miRNA mimics to targeting miRNAs in cancer have been conducted and demonstrated that potential for developing novel therapeutic methods^7^.

FAS, also known as CD95, was discovered as an apoptosis-inducing receptor^12^. Activated FAS recruits FAS-associated protein with death domain (FADD). FADD further recruits caspase-8, caspase-10, and cellular FADD-like interleukin-1-β-converting enzyme-inhibitory protein (c-FLIP) to form the death-inducing signaling complex (DISC)^13^, which mediates both apoptotic and non-apoptotic signaling pathways^14^. Accumulated evidence has indicated that FAS plays a key role in cancer progression^12^. Activated FAS signaling suppresses lung cancer cell growth in a mouse model through increasing TH9 cell differentiation^15^. In contrast, the FAS pathway facilitates colon cancer growth and metastatic potential by inducing the Erk1/2 pathway^16^. In addition, it has been reported that the miR-23a/b^17^ and miR-106a^18^ could target FAS to promote thymic lymphoma and gastric cancer progression, respectively.

In this study, we found that *miR-196b-5p* plays oncogenic functions by directly targeting FAS. FAS expression was significantly negatively correlated with the *miR-196b-5p* expression in NSCLC. Knocking down FAS promoted lung cancer cells growth through NFkB activation-mediated enhanced IL6 secretion and subsequent STAT3 activation. Our results demonstrated that the *miR-196b-5p*/FAS/NFkB/IL6/STAT3 axis might be a potential therapeutic target in NSCLC.

## Materials and methods

### Cell culture and reagents

The human lung cancer cell lines (A549, H460, and H292) were purchased from the American Type Culture Collection (ATCC). A549, H460, and H292 cells were cultured in RPMI-1640 medium (Gibco, Carlsbad, CA, USA) supplemented with 10% fetal bovine serum (FBS) (Gibco, Carlsbad, CA, USA) and 1% penicillin–streptomycin (Gibco, Carlsbad, CA, USA). Cell Cycle Regulation Antibody Sampler Kit (9932), anti-p65 antibody (8242), and anti-GAPDH rabbit monoclonal antibody (5174) were purchased from Cell Signaling Technology. Antibodies against p-STAT3 (Y705) (ab76315), STAT3 (ab68153), and Lamin B1 (ab16048) were purchased from Abcam. Anti-FAS/CD95 rabbit polyclonal antibody (13098–1-AP) was obtained from Proteintech, and an anti-vinculin antibody was ordered from Sigma-Aldrich. The miR-196b mimics, siFAS, and siSTAT3 were purchased from Genepharma (Shanghai, China). p65 overexpressing plasmid was kindly provided from Hui-lung Sun (Chicago University).

### Transfection

Transfection of siRNAs against FAS, STAT3, and negative control was carried out in Lipofectamine 3000 (Invitrogen, Carlsbad, CA, USA) according to the manufacturer’s instruction (Invitrogen). p65 overexpressing vector, control vector, miR-196b mimic, and control mimic were transfected to the cells by using Lipofectamine 3000 (Invitrogen, Carlsbad, CA, USA) according to the manufacturer’s instruction (Invitrogen). The sequences of siRNAs are shown in Supplementary Table S1.

### RNA extraction and quantitative real-time PCR

Total RNAs were extracted by TRIzol Reagent (Invitrogen, Carlsbad, CA, USA) according to the manufacturer’s instruction. For cDNA synthesis, 1 μg of the total RNA was transcribed using the PrimeScript™ RT reagent Kit with gDNA Eraser (TAKARA, Japan) according to the manufacturer’s instruction. Quantitative real-time PCR was performed by using TB Green Fast qPCR mix (TAKARA, Japan) according to the manufacturer’s instruction. All reactions were conducted in triplicates. RT-PCR primers for *IL6*, *FAS*, and *GAPDH* are shown in Supplementary Table S2.

### Cell proliferation assays

Two or three thousand cells in RPMI-1640 medium with 10% FBS were added to each well in a 48-well plate in sextuplicate and cultured at 37 °C for 4 days. The viability of cells was determined by MTT assay. To investigate the FAS knockdown or p65 overexpression-induced IL6 secretion on lung cancer cell proliferation, FAS knockdown or p65 overexpression lung cancer cells were cultured in six-well plates at 5 × 10^5^ cells/well and incubated in RPMI-1640 medium with 2% FBS overnight. The cells were washed with PBS, and 2 ml of serum-free medium were added to each well. After 24 h, conditioned medium were collected and subjected to in vitro cell proliferation assay.

### Cell colony-formation assay

After 24 h transfection, 500–1000 cells in RPMI-1640 medium with 10% FBS were added to each six-well plate in triplicate, and cultured at 37 °C for 6–9 days. Cells were fixed with 4% paraformaldehyde in PBS for 15 min, and stained with crystal violet (Beyotime, China). The colony number was calculated by Image J software.

### Western blot analysis

The cells were lysed with RIPA buffer (Boster, China) supplemented with protease/phosphatase inhibitor Cocktail (Boster, China). Proteins from the lysate were separated by electrophoresis using 10–12% polyacrylamide gels and electrotransferred to PVDF membranes (Bio-Rad). After blocking with 5% skimmed milk, membranes were incubated with various primary antibodies. Then membranes were incubated with appropriate horseradish peroxidase (HRP)-conjugated secondary antibody. Specific proteins were detected with EZ-ECL Kit (Biological Industries).

### Immunohistochemistry

The harvested tumor tissues were fixed with 10% formalin, processed, and embedded in paraffin. In total, 5-µm-thick sections were placed on positively charged slides. The tissue sections were stained by routine immunohistochemical techniques and incubated with primary antibodies against FAS (1:250) overnight at 4 °C. Conjugated secondary antibodies and diaminobenzidine (DAB) were used to detect FAS before hematoxylin staining and a neutral gum sealing. The FAS positive cells were counted and photographed with an ortho-microscope (Laica, Germany). According to the percentage of positive cells in the mean average of five fields, immunohistochemical reactivity for FAS was scored as follows: 0–5% (−), 5–25% (1+), 25–50% (2+), 50–100% (3+). The FAS expression graded as 3+ was defined as strong, 2+ was defined as moderate, and 1+ was defined as weak, respectively.

### Flow-cytometry analysis for cell cycle

The cells were fixed with 70% ethanol at −20 °C overnight, then washed with PBS and resuspended in BD Pharmingen™ PI/RNase Staining Buffer (BD Biosciences). The cells were incubated at 37 °C for 40 min, and the cell cycle was detected by FACS Calibur Flow Cytometer (BD Biosciences). The results were processed by FlowJo10 software.

### ELISA assay

To investigate IL6 secretion levels in media, miR-196b overexpression, FAS knockdown, or p65 overexpression, lung cancer cells were cultured in six-well plates at 5 × 10^5^ cells/well and incubated in RPMI-1640 medium with 2% FBS overnight. The cells were washed with PBS, and 2 ml of serum-free medium were added to each well. After 24 h, suspensions of medium were collected and subjected to enzyme-linked immunosorbent assay (ELISA). IL6 secretion level in suspensions was detected according to the manufacturer’s instruction of IL6 Human Uncoated ELISA Kit (Invitrogen).

### Cytosol and nuclear protein extraction

Cells were harvested and lysed using Nuclear and Cytoplasmic Protein Extraction Kit (Beyotime, China) according to the manufacturer’s instruction. Protein expression of the cytoplasmic and nuclear extractions was determined by western blot analysis. Lamin B was used as a marker of nuclear protein, and GAPDH was used as a marker of cytoplasmic protein.

### Patient samples

This study was approved by the Institutional Research Human Ethical Committee of the Wenzhou Medical University for the use of clinical biopsy specimens, and informed consent was obtained from the patients. A total of 30 NSCLC tissues and 30 corresponding NATs were obtained postoperatively from Zhoushan Hospital of Wenzhou Medical University. NSCLC tissues and NATs from patients were immediately placed in liquid nitrogen and then stored at −80 °C until further analysis.

### TCGA dataset

The TCGA miRNA-seq and RNA-seq data with clinical information were downloaded on July 31, 2013. Only log-2 transformed level 3 data were used for analysis. For analysis of the TCGA dataset, the Welch *t* test was conducted to determine if the *miR-196b-5p* expression is different between NSCLC tissues and NATs. For the correlation analysis between *miR-196b-5p* expression and *FAS* expression, Pearson correlation coefficients were calculated.

### Statistical analysis

All statistical analyses were performed by GraphPad Prism 7.0 (GraphPad Software, La Jolla, CA, USA). Data are represented as means with standard deviation (SD), and statistical significance was determined with unpaired *t* tests unless indicated otherwise. *P* values < 0.05 were considered statistically significant.

## Results

### Upregulated *miR-196b-5p* inhibits FAS expression in NSCLC

**Fig. 1:**
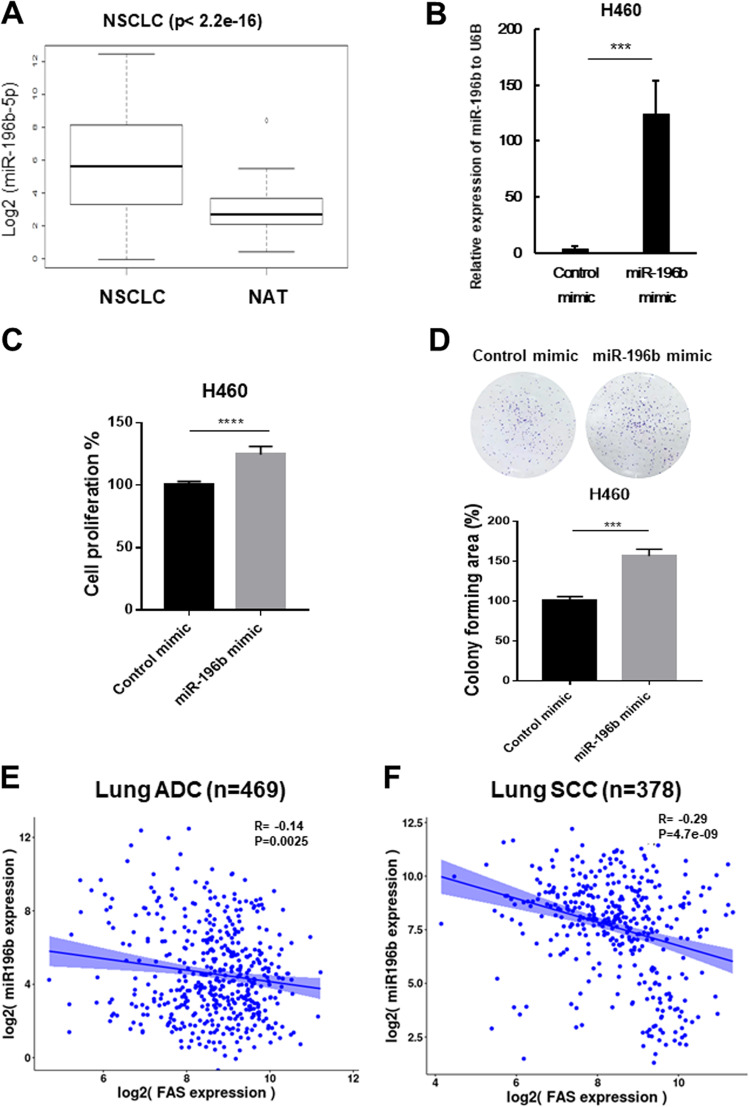
*miR-196b-5p* plays an oncogenic function in lung cancer cells and is negatively correlated with *FAS* in non-small cell lung cancer (NSCLC). **a** *miR-196b-5p* expression data were obtained from the TCGA miR-seq dataset. Of evaluable 666 NSCLC patients, 86 patients had matched normal adjacent tissues. **b** qRT-PCR examines *miR-196b-5p* expression in *miR-196b-5p* overexpressing H460 cells and control cells. Results are represented as means ± SD (*n* = 3). **c** Cell proliferation assay for *miR-196b-5p* overexpressing H460 cells and control cells. Results are represented as means ± SD (*n* = 3). **d** Colony-formation assay for *miR-196b-5p* overexpressing H460 cells and control cells. Colony-forming areas were measured by Image J software, and relative colony-forming areas were calculated by comparing with corresponding controls. Results are represented as means ± SD (*n* = 3). **e**, **f** *FAS* expression from TCGA RNA-seq data and *miR-196b-5p* expression from miR-seq data were used to examine the correlation between *miR-196b-5p* and *FAS* expressions in lung ADC dataset (*n* = 469) (**e**) and lung squamous cell carcinoma (SCC) dataset (*n* = 378) (**f**). ***P < 0.001, ****P < 0.0001.

Recently, we found that *miR-196b-5p* functions as an oncomiRNA in NSCLC by directly targeting GATA6 and TSPAN12^19^. To further validate the oncogenic function of *miR-196b-5p* in NSCLC, we analyzed *miR-196b-5p* expression in NSCLC tissues and normal adjacent tissues (NATs) using The Cancer Genome Atlas (TCGA) dataset. We found that higher *miR-196b-5p* expression in NSCLC tissues than those in NATs (Fig. 1a). In detail, the average expression level of *miR-196b-5p* in NSCLC tissues is about ten times higher than those in NATs. Of note, almost 100 times higher *miR-196b-5p* expression was observed in certain NSCLC tissue samples than the average expression level of *miR-196b-5p* in NATs. Overexpressing *miR-196b-5p* in H460 cells (Fig. 1b) promoted cell proliferation (Fig. 1c) and colony formation (Fig. 1d). Similar results were also observed in *miR-196b-5p* overexpressing H292 cells (Supplementary Fig. 1A–C) and H520 cells (Supplementary Fig. 1D–F). Our recent study suggested that FAS might be a potential target of *miR-196b-5p* in NSCLC^20^. Bioinformatics analyses showed that five of seven miRNA target prediction websites indicated that FAS is a direct target of *miR-196b-5p* (Supplementary Table S3). It has also been reported that FAS might be a target of *miR-196b* in colorectal cancer^20^ and mixed-lineage leukemia (MLL)-rearranged leukemia^21^. Thus, we further examined the correlation between *miR-196b-5p* and *FAS* in NSCLC. TCGA lung adenocarcinoma (ADC) (*n* = 469) samples and lung SCC (*n* = 378) samples having both *miR-196b-5p* and *FAS* expression data available were selected for Pearson correlation analysis. The results showed that *miR-196b-5p* was negatively correlated with *FAS* in both lung ADC (*R* = −0.14, *P* = 0.0025) and lung SCC (*R* = −0.29, *P* = 4.7e-09), suggesting that reduced expression of *FAS* might be related to the upregulation of *miR-196b-5p* in NSCLC (Fig. 1e, f).

**Fig. 2:**
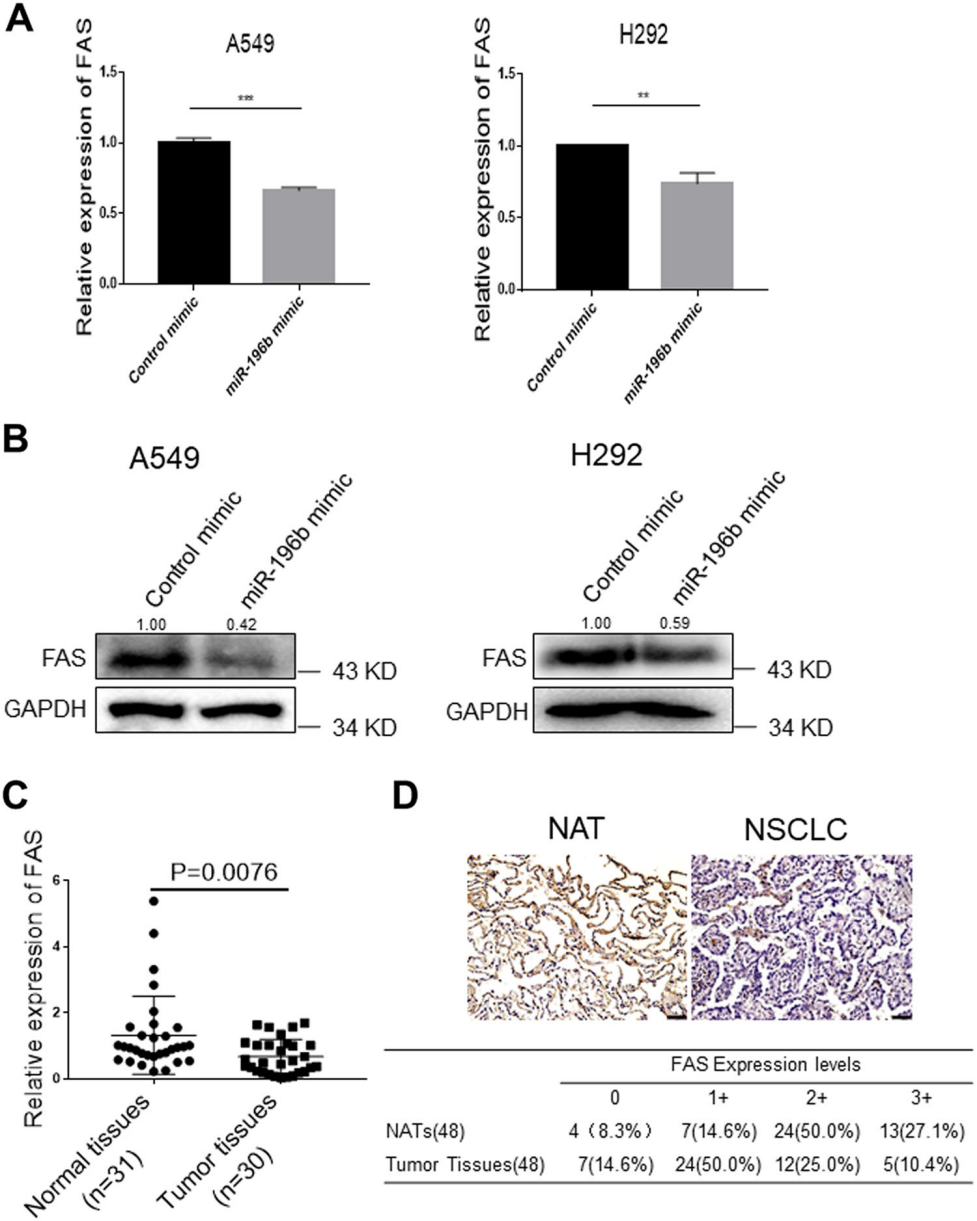
FAS is the target of *miR-196b-5p* and is downregulated in non-small cell lung cancer (NSCLC). **a** *FAS* expression in A549 and H292 lung cancer cells after overexpressing *miR-196b-5p* was determined by qRT-PCR. Results are represented as means ± SD (*n* = 3). **b** Western blot analysis of FAS protein in A549 and H292 lung cancer cells after overexpressing *miR-196b-5p*. The bands were quantified using Image J software, and relative values were obtained by normalizing to the value of each corresponding GAPDH. **c** Expression level of *FAS* in 60 paired NSCLC tissues and their matched NATs. The RNA samples were extracted from 30 NSCLC tissues and 30 corresponding NATs. The RNAs were subject to qRT-PCR with a *FAS* probe, and the expression was normalized by *GAPDH*. **d** Upper panel shows representative immunohistochemical staining for FAS in NSCLC tissues and normal adjacent tissues (NATs) from the same patient. Summary of tissue immunohistochemical staining data for FAS in 48 pairs of clinical NSCLC tissues and NATs are shown in the lower panel. **P < 0.01, ***P < 0.001.

Next, to investigate the effects of *miR-196b-5p* on the expression of FAS mRNA and protein, we transiently overexpressed *miR-196b* mimics in A549 and H292 lung cancer cells. Overexpressing *miR-196b-5p* markedly reduced FAS mRNA (Fig. 2a) and protein (Fig. 2b) levels in both A549 and H292 cells, suggesting that *miR-196b-5p* might directly target FAS and enhanced expression of *miR-196b-5p* may reduce FAS expression in NSCLC.

### FAS expression is downregulated in NSCLC

Several studies have reported that FAS expression was downregulated in various cancers, including lung cancer^22–24^. Viard-Leveugle et al. has shown that FAS expression was markedly decreased in ~90% of lung cancer tissue samples compared with the normal lung tissue samples, and 24% of lung cancer tissue samples were completely lost FAS expression^24^. Since *miR-196–5p* was negatively correlated with *FAS* expression, we further evaluated *FAS* expression in NSCLC using TCGA lung ADC and SCC dataset. Analysis of *FAS* expression in 334 lung ADC patients with 57 matched NATs (Supplementary Fig. 2A) and 349 lung SCC patients with 51 matched NATs (Supplementary Fig. 2B) showed that significantly downregulated FAS expression in both lung ADC (*P* = 2.72e-14) and SCC (*P* = 7.92e-28). We further analyzed *FAS* expression in 30 NSCLC tissues and 31 NATs from Zhoushan Hospital of Wenzhou Medical University. Consistent with TCGA dataset and previous study, we found that *FAS* expression was downregulated in NSCLC tissues compared with those in NATs (*P* = 0.0076) (Fig. 2c). In addition, we analyzed FAS protein expression levels in tissue samples from 48 NSCLC patients by immunohistochemical staining. We found that FAS immunoreactivity was markedly reduced in NSCLC tissue samples compared with those in NATs samples. Of note, 77.1% of the NAT specimens showed moderate-to-strong FAS expression; however, only 35.4% of NSCLC tissue specimens presented moderate-to-strong FAS expression (Fig. 2d). Kaplan–Meier survival analysis using 1882 available NSCLC patients from the Kaplan–Meier Plotter showed that high expression of *FAS* is significantly associated with favorable prognosis of NSCLC patients (HR = 0.77, *P* = 6.5e-05) (Supplementary Fig. 2C). These results indicate that both mRNA and protein levels of FAS were downregulated in NSCLC tissues, and FAS may play an essential role in NSCLC carcinogenesis. Considering FAS mediates external cell death signaling, we speculate that manipulation of FAS expression without external cell death signal may not impact on the cell death rate of lung cancer cells.

### FAS knockdown promotes lung cancer cell proliferation and cell cycle

**Fig. 3:**
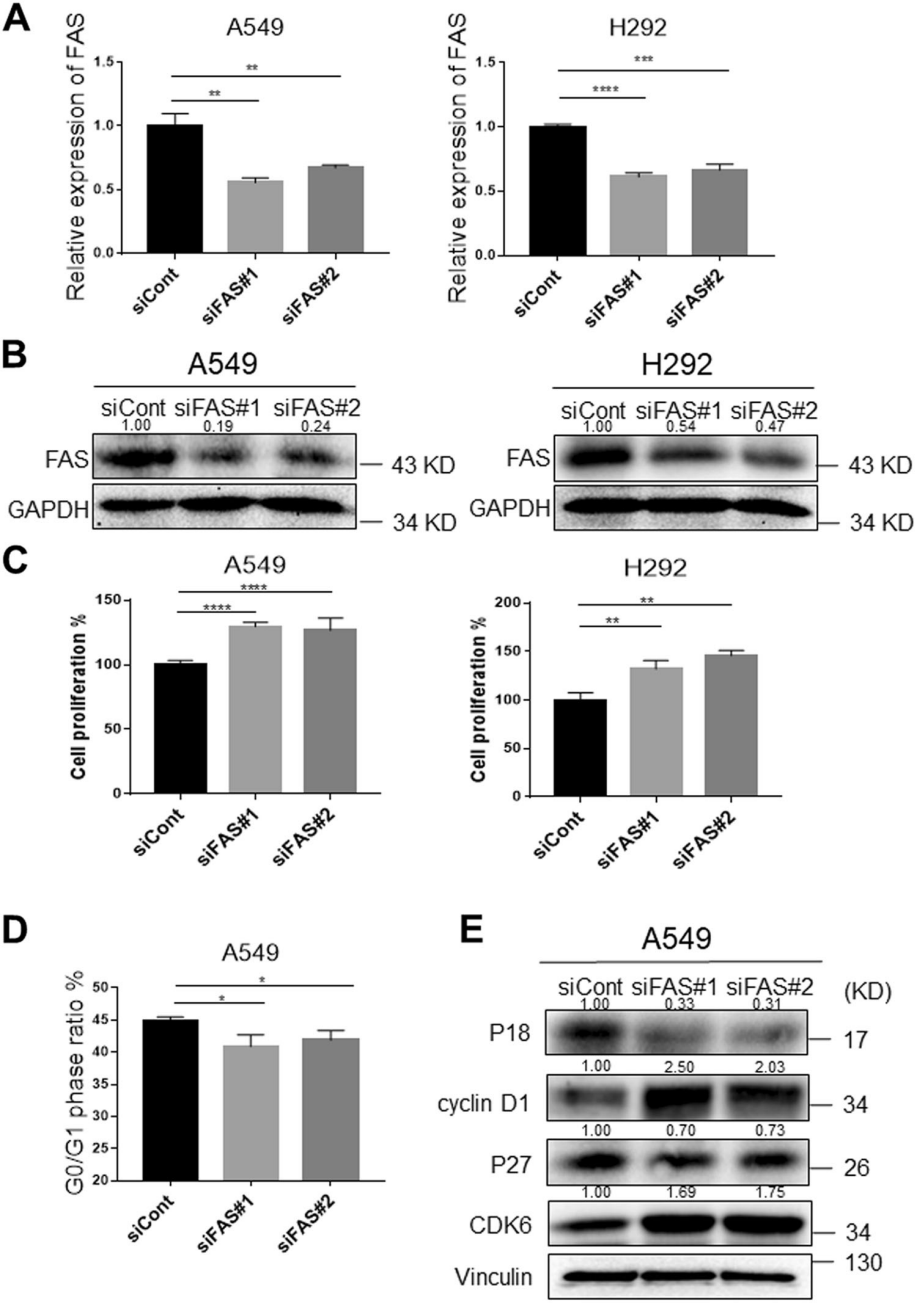
FAS knockdown promotes lung cancer cell proliferation and cell cycle. **a** qRT-PCR measure *FAS* expression in FAS knockdown A549 and H292 lung cancer cells. The expression was normalized by *GAPDH*. Results are represented as means ± SD (*n* = 3). **b** Western blot analyses of FAS protein in FAS knockdown A549 and H292 lung cancer cells. The bands were quantified using Image J software, and relative values were obtained by normalizing to the value of each corresponding GAPDH. **c** Cell proliferation assay for A549 and H292 cells after transfection with siFAS for 4 days. Results are represented as means ± SD (*n* = 3). **d** The percentage of cell cycle G1 phase cells in FAS knockdown A549 lung cancer cells and control cells. Results are represented as means ± SD (*n* = 3). **e** Western blot analyses for cell cycle G1 phase-related proteins in FAS knockdown A549 lung cancer cells and control cells. The bands were quantified using Image J software, and relative values were obtained by normalizing to the value of each corresponding Vinculin. *P < 0.05, **P < 0.01, ***P < 0.001, ****P < 0.0001.

To further investigate the functions of FAS in lung cancer cells proliferation, colony formation, and cell cycle, we knocking down FAS in A549 and H292 cells by two independent siRNAs since both cell lines have relatively higher *FAS* expression than other lung cancer cell lines (Supplementary Fig. 3A). The FAS mRNA (Fig. 3a) and protein (Fig. 3b) expression levels after knockdown were confirmed by qRT-PCR analysis and western blot analysis. Knocking down FAS (~50%) significantly promoted cell proliferation (Fig. 3c) and colony formation (Supplementary Fig. 3B, C) in both A549 and H292 cells. We further investigated the effects of FAS knockdown on an SCC cell line, H520 cells. As expected, knocking down FAS enhanced the proliferative and colony-forming ability of H520 cells (Supplementary Fig. 3D, E). In addition, cell cycle analysis showed that knocking down FAS promoted transition of the G1 to S cell cycle in both A549 and H292 cells (Fig. 3d and Supplementary Fig. 4A, B). Accordingly, cell cycle G1 phase-related proteins were consistently changed in both A549 and H292 cells after knocking down FAS, including cyclin D1, CDK6, CDK inhibitor 1B (P27), and 2C (P18) (Fig. 3e and Supplementary Fig. 4C). These findings indicated that increased lung cancer cell proliferative and colony-forming ability by FAS knockdown might be due to accelerated cell cycle.

### FAS knockdown activates STAT3 signaling

**Fig. 4:**
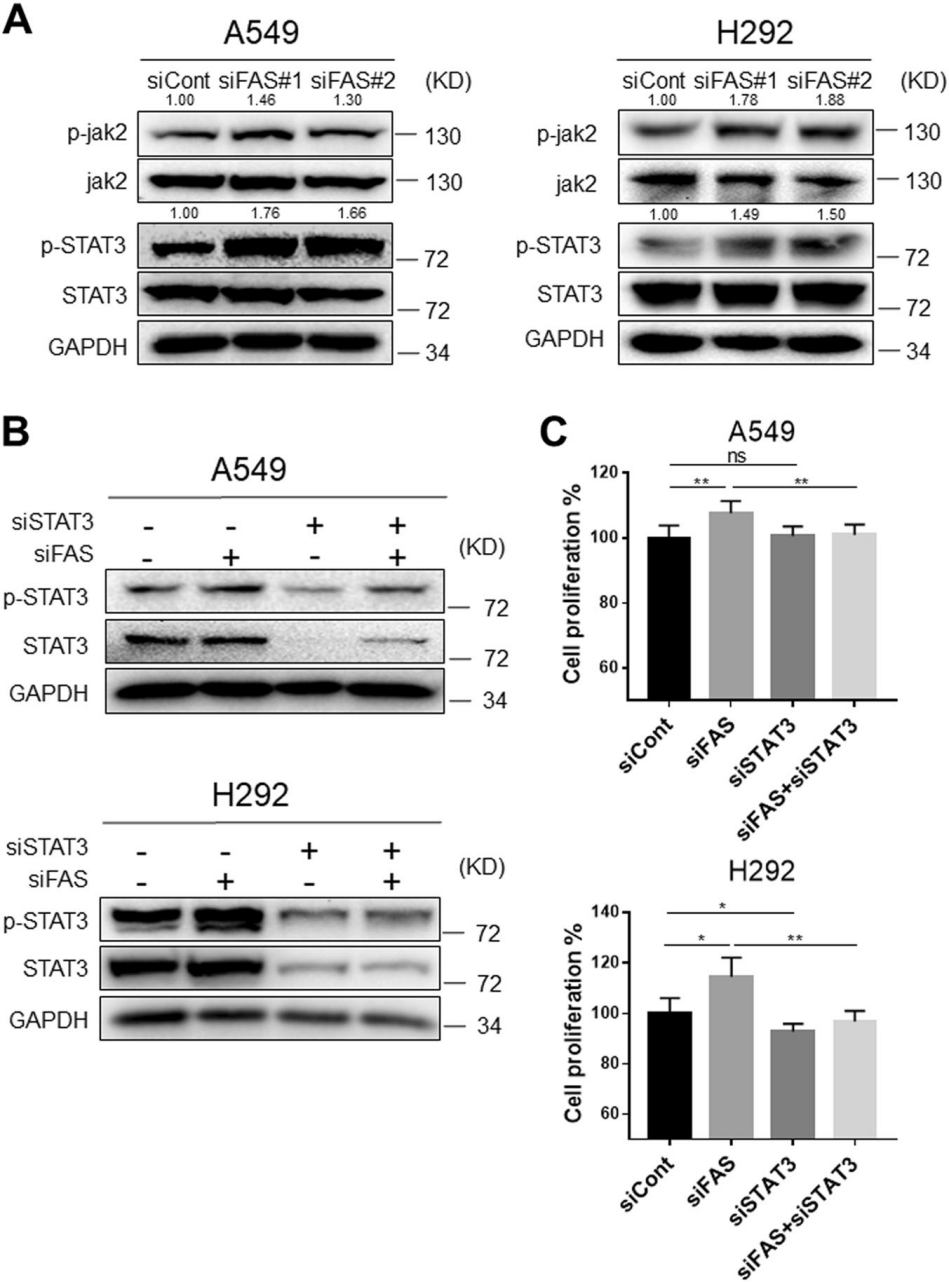
FAS knockdown promotes STAT3 phosphorylation. **a** Western blot analyses for p-STAT3 (Y705), STAT3, p-Jak2, and Jak2 in FAS knockdown A549 and H292 lung cancer cells. The bands of p-STAT3 and p-Jak2 were quantified using Image J software, and relative values were obtained by normalizing to the value of each corresponding STAT3 and Jak2. **b** Western blot analysis to detect p-STAT3 (Y705) expression in FAS knockdown A549 and H292 lung cancer cells transfecting with or without STAT3 siRNAs. **c** Cell proliferation assay in FAS knockdown A549 and H292 lung cancer cells transfecting with or without STAT3 siRNAs. Results are represented as means ± SD (*n* = 3). *P < 0.05, **P < 0.01, ns no significance.

FAS signaling is closely associated with the immunological and inflammatory pathway^25^. Accumulating evidence suggests that FAS regulates T cells differentiation by in combination with STATs family proteins^26^. Particularly, STAT3 is highly activated in various cancers, and activated STAT3 promotes cancer progression^27^. It has been reported that STAT1/3-Mcl-1 signaling regulates FAS-mediated apoptosis in solid cancers^28^, and anti-FAS antibody-mediated activation of FAS could increase the phosphorylated STAT3 expression level in gastric cancer^29^. To investigate whether FAS signaling is involved in STAT3 activation in NSCLC, we performed a series of knockdown experiments. Knocking down FAS consistently enhanced phosphorylation levels of jak2 and STAT3 in both A549 and H292 cells (Fig. 4a). Similar results were also observed in FAS knocking down H520 cells (Supplementary Fig. 3F). To further demonstrate the relationship between STAT3 and FAS in NSCLC, we knocking down STAT3, FAS, or both of them in lung cancer cells. As expected, knocking down FAS increased STAT3 phosphorylation, while knocking down STAT3 reduced the levels of total STAT3 and phosphorylated STAT3. Moreover, we found that knocking down both STAT3 and FAS consistently attenuated FAS-knockdown-induced STAT3 phosphorylation (Fig. 4b). Accordingly, increased proliferative (Fig. 4c) and colony-forming abilities (Supplementary Fig. 4D) by FAS knockdown were significantly attenuated by knocking down both STAT3 and FAS. Together, these results suggested that the FAS downregulation promotes lung cancer cell growth by activating the STAT3 signaling pathway.

### FAS knockdown facilitates p65 nuclear translocation and promotes IL6 transcription

**Fig. 5:**
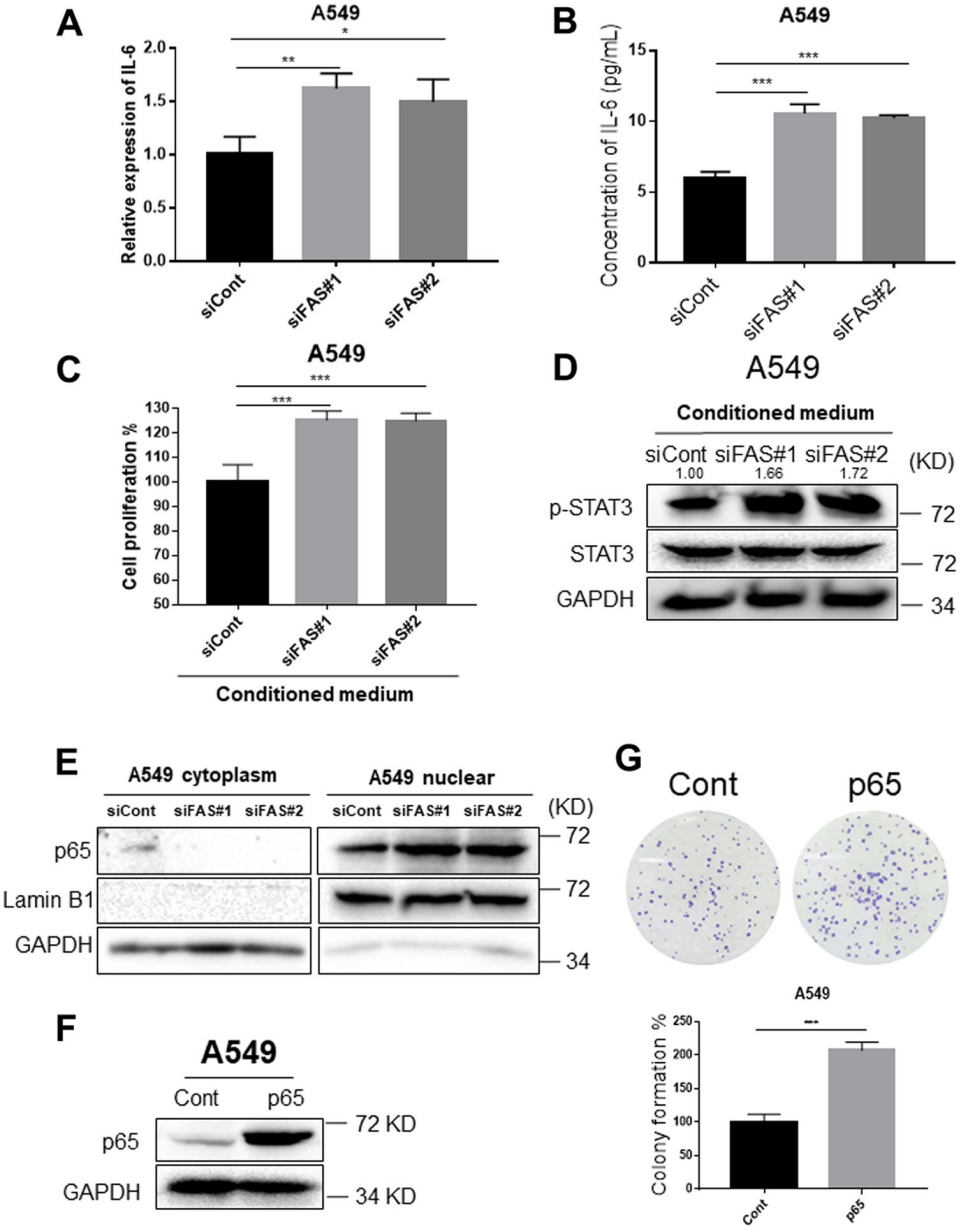
FAS knockdown enhances IL6 expression. **a** qRT-PCR measure *IL6* expression in FAS knockdown A549 lung cancer cells. The expression was normalized by *GAPDH*. Results are represented as means ± SD (*n* = 3). **b** ELISA measures secreted IL6 protein levels in conditioned media from FAS knockdown A549 cells and control cells. Results are represented as means ± SD (*n* = 3). **c** Cell proliferation assay of A549 cells treated with conditioned mediums from FAS knockdown lung cancer cells or control cells. Results are represented as means ± SD (*n* = 3). **d** Western blot analysis for p-STAT3 (Y705) expression in A549 cells treated with conditioned mediums from FAS knockdown lung cancer cells or control cells. The p-STAT3 bands were quantified using Image J software, and relative values were obtained by normalizing to the value of each corresponding STAT3. **e** Western blot analyses for p65 protein in FAS knockdown A549 cells and control cells. Both nuclear and cytosol proteins were extracted and subjected to western blot analysis. **f** Western blot analyses of p65 protein in p65 overexpressing A549 cells and control cells. **g** Colony-formation assay for p65 overexpressing A549 cells and control cells. Colony-forming areas were measured by Image J software, and relative colony-forming areas were calculated by comparing with corresponding controls. Results are represented as means ± SD (*n* = 3). *P < 0.05, **P < 0.01, ***P < 0.001.

Our results showed that the FAS knockdown activated STAT3 signaling. Increased IL6 expression has been reported to activate STAT3 signaling, subsequently involving drug resistance and cancer progression^30^. Accumulating evidence has suggested that FAS facilitates the secretion of proinflammatory cytokines and chemokines, including CXCL1/KC, CXCL2/MIP2, IL6, and IL8^31–33^. Among them, IL6 is a well-characterized cytokine that has been reported to be involved in various cancers progression by stimulating JAK2/STAT3 pathway^30^. Since both FAS and IL6 were closely associated with cancer pathogenesis and STAT3 activation, we investigated IL6 mRNA and protein levels after FAS knockdown. IL6 mRNA (Fig. 5a) and protein (Fig. 5b) expression was significantly increased in FAS knocking down A549 cells compared to the control cells. As expected, knockdown of FAS in H520 cells significantly enhanced IL6 protein secretion compared to the control cells (Supplementary Fig. 5D). Based on this result, we speculate that FAS knockdown might promote IL6 secretion from NSCLC cells.

To verify this hypothesis, conditioned medium from FAS knockdown and control cells were subjected to cell proliferation and colony-formation assays (Supplementary Fig. 5A). Conditioned medium from FAS knockdown cells significantly enhanced cell proliferative ability (Fig. 5c) and colony-forming ability compared with those conditioned media from control cells (Supplementary Fig. 5C). Interestingly, conditioned medium from FAS knockdown cells markedly increased STAT3 phosphorylation (Fig. 5d), suggesting that increased IL6 expression by FAS knockdown is responsible for STAT3 activation in lung cancer cells. p65, a member of the NFkB family, is a well-known transcription factor that has been reported to enhance IL6 transcription by binding its promoter region^34,35^. Hence, we checked whether p65 was activated following FAS knockdown. Indeed, FAS knockdown promoted nuclear translocation of p65 protein compared to those control cells, suggesting that NFkB activation by FAS knockdown is the main cause of increased IL6 expression in lung cancer cells (Fig. 5e).

### NF-κB-mediated enhancement of IL6 expression promotes STAT3 activation

**Fig. 6:**
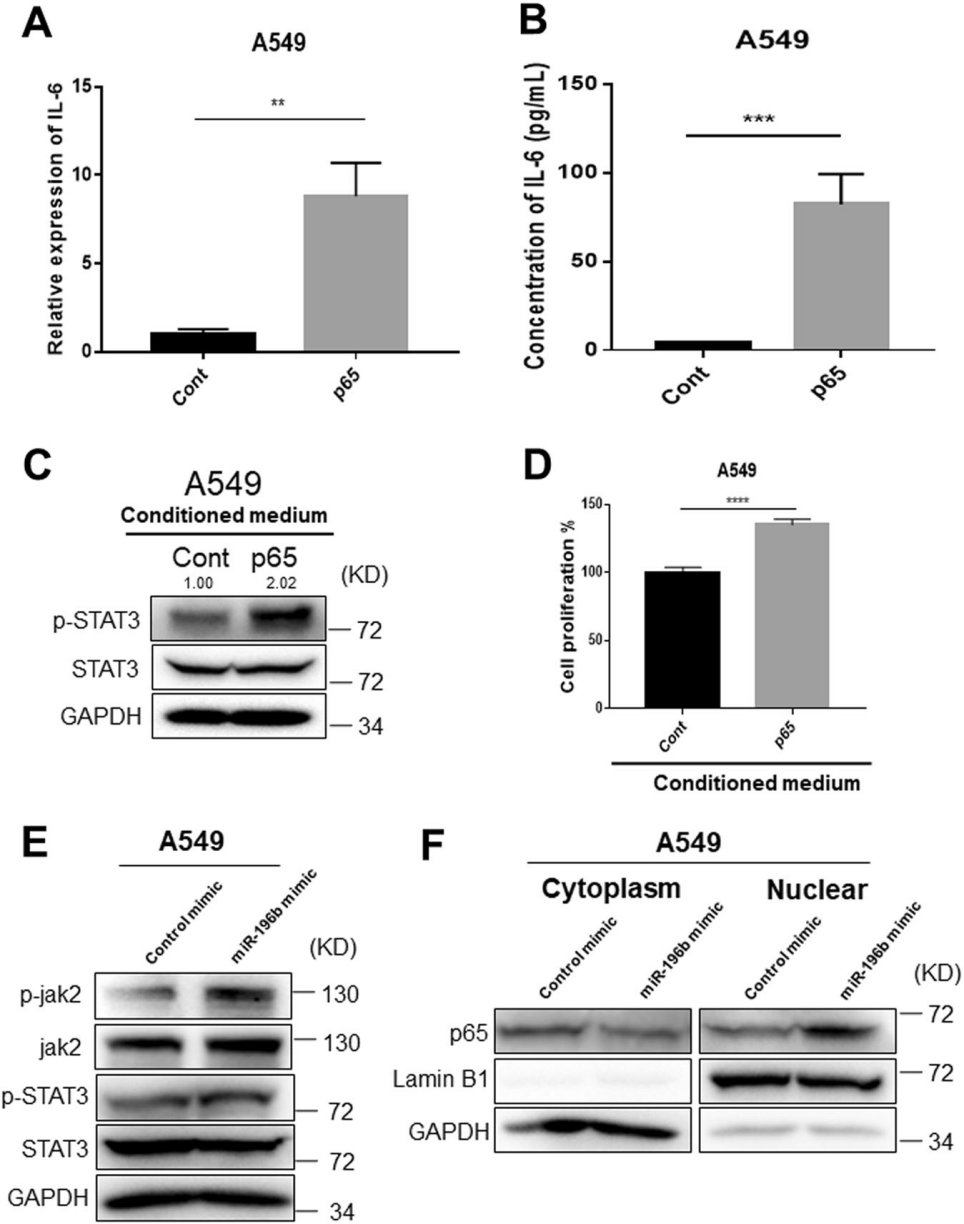
FAS knockdown-mediated p65 activation promotes IL6 expression. **a** qRT-PCR measure *IL6* expression in p65 overexpressing A549 cells and control cells. The expression was normalized by *GAPDH*. Results are represented as means ± SD (*n* = 3). **b** ELISA measures secreted IL6 protein levels in conditioned media from p65 overexpressing A549 cells and control cells. **c** Western blot analysis for p-STAT3 (Y705) expression in A549 cells treated with conditioned mediums from p65 overexpressing lung cancer cells or control cells. The p-STAT3 bands were quantified using Image J software, and relative values were obtained by normalizing to the value of the corresponding STAT3. **d** Cell proliferation assay of A549 cells treated with conditioned mediums from p65 overexpressing lung cancer cells or control cells. Results are represented as means ± SD (*n* = 3). **e** Western blot analyses for p-STAT3 (Y705), STAT3, p-Jak2, and Jak2 in miR-196b-5p overexpressing lung cancer cells and control cells. **f** Western blot analyses for p65 protein in miR-196b-5p overexpressing A549 cells and control cells. Both nuclear and cytosol proteins were extracted and subjected to western blot analysis. **P < 0.01, ****P < 0.0001.

To further demonstrate the relationship between p65, IL6, and STAT3 signaling, we overexpressed p65 in lung cancer cells. Increased p65 protein expression after overexpressing was confirmed by western blot analysis (Fig. 5f). Overexpressing p65 significantly enhanced colony-forming ability of lung cancer cells (Fig. 5g). Since NFkB signaling could regulate IL6 expression in inflammation and cancer^35,36^, we further investigated IL6 expression in p65 overexpressing lung cancer cells. As expected, p65 overexpression significantly increased IL6 mRNA and protein expressions (Fig. 6a, b). In addition, conditioned medium from p65 overexpressing A549 cells markedly enhanced phosphorylated STAT3 expression, subsequently promoted cell proliferation in A549 cells compared with those conditioned medium from control cells (Fig. 6c, d, and Supplementary Fig. 5B). Pearson correlation analyses using TCGA NSCLC dataset from GEPIA website showed that *p65* was positively correlated with both *IL6* (*P* < 2.2e-16, *R* = 0.2) and *STAT3* (*P* < 2.2e-16, *R* = 0.41) (Supplementary Fig. 6A, B). A positive correlation between *IL6* and *STAT3* (*P* = 1.3e-11, *R* = 0.2) was also observed (Supplementary Fig. 6C). Considering low *R* values for correlation between IL6 and P65 or STAT3, further studies need to be done to validate these results. We further investigated the effects of *miR-196b-5p* overexpression on the expression levels of phospho-STAT3 and nuclear translocation of p65. Overexpression of *miR-196b-5p* slightly enhanced phospho-STAT3 expression (Fig. 6e); however, consistently increased nuclear translocation of p65 (Fig. 6f). Furthermore, *miR-196b-5p* significantly upregulated IL6 mRNA expression (Supplementary Fig. 7A); however, no significant difference was observed for secreted IL6 protein levels (Supplementary Fig. 7B). These results suggested that *miR-196b-5p*-mediated FAS downregulation at least partly contributes to the activation of p65-IL6-STAT3 signaling, subsequently promotes NSCLC progression.

## Discussion

Dysregulated miRNA expression is frequently seen in cancer. In cancer, miRNA may function as an oncogene or tumor suppressor, depending on cancer tissue type^37^. *miR-196b* plays dual functions in cancer; however, its role in lung cancer remains controversial. Our recent study showed that QKI-5 negatively regulates *miR-196b-5p*, and upregulated *miR-196b-5p* promotes lung cancer cell migration, proliferation, and cell cycle through directly targeting the tumor suppressors, GATA6 and TSPAN12 in NSCLC^19^. In addition, *miR-196b-5p* has been reported to function as an oncogene in laryngeal SCC by targeting SOCS2^38^, and in oral cancer through regulating the NME4-JNK-TIMP1-MMP signaling pathway^39^. *miR-196b* simultaneously targets both HOXA9 and MEIS1 oncogenes, and FAS tumor suppressor to regulate tumorigenesis in MLL-rearranged leukemia^21^. In our study, we found that upregulated *miR-196b-5p* promotes lung cancer cell growth by targeting known tumor suppressor, FAS, in NSCLC. The expression of FAS is negatively correlated with the expression of *miR-196b-5p*, and is markedly downregulated in NSCLC tissues. Further analysis showed that high expression of FAS is associated with a favorable prognosis of NSCLC. Knocking down FAS promoted lung cancer cell growth by accelerating cell cycle, suggesting that bona fide tumor-suppressive functions of FAS in NSCLC.

The STAT family proteins were discovered as the transcription factors that bind to specific DNA sequences in the promoter region regulating gene transcription^40^. Persistent STAT3 activation promotes tumor progression and metastasis in various cancer^27,41^. FAS signaling facilitates apoptotic cell death in lung cancer cells, and advanced lung cancer is associated with decreased expression of FAS in circulating CD8 + T cells^42,43^. In contrast, it has been reported that FAS signaling promotes gastric cancer progression by activating STAT3 signaling^29^. Tyrosine phosphorylation of FAS (pY-291-FAS) activates STAT3, and subsequent AKT and MAPK signaling pathway resulting in increased cell proliferation and migration^44^, suggesting that dual functions of FAS in cancer progression. Nevertheless, the underlying molecular mechanisms of FAS in NSCLC progression, particularly linking between FAS and STAT3 signaling still remain unclear. Here, we found that FAS knockdown consistently activates the JAK2/STAT3 pathway. Since IL6 could promote STAT3 signaling pathway^30,35^, we further evaluated IL6 expression after knocking down FAS in lung cancer cells. As expected, we observed that increased IL6 expression in FAS knockdown lung cancer cells. Activated NFkB plays a key role in various cancer progression and could enhance IL6 secretion in tumor microenvironment^36,45^. To further demonstrate underlying molecular mechanisms of FAS knockdown-mediated enhancement of IL6 expression, we investigated p65 protein nuclear translocation in FAS knockdown lung cancer cells. Knocking down FAS indeed increased nuclear p65 expression, indicating that FAS knockdown induced IL6-mediated STAT3 activation might be due to activated NFkB signaling in NSCLC. Furthermore, we confirmed that overexpressing p65 elevated IL6 expression, and subsequent STAT3 activation resulting in enhanced lung cancer cell growth rate. Although a direct link between *miR-196b-5p* and phospho-STAT3 or IL6 is weak, FAS knockdown-mediated activation of p65-IL6-STAT3 is consistent in our study. We think that a number of downstream targets of miR-196b and their crosstalk might attenuate the effects of *miR-196b-5p* on the expression of phospho-STAT3 and IL6. Further study needs to validate this hypothesis. Our study revealed for the first time that FAS knockdown facilitates STAT3 activation in lung cancer cells. FAS knockdown accelerated nuclear translocation of p65, which in turn promotes IL6 expression and subsequent STAT3 phosphorylation.

**Fig. 7:**
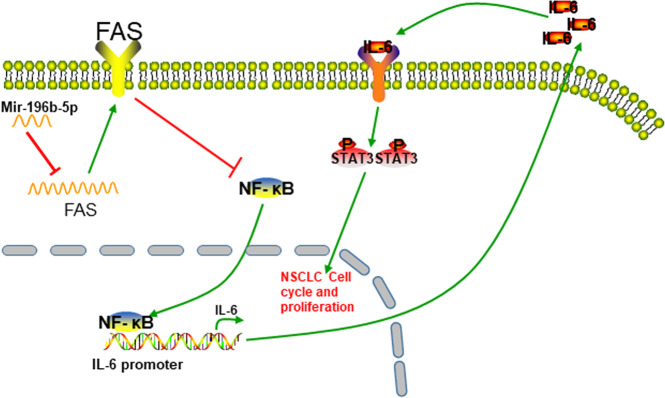
Schematic illustration of summary of this study. Proposed dysregulated *miR-196b-5p-*mediated downregulation of FAS activates STAT3 signaling through the NFkB-IL6 axis in NSCLC.

Our current study revealed that *miR-196b-5p*-mediated downregulation of FAS might involve activation of STAT3 signaling through the NFkB-IL6 axis (Fig. 7). The study provided important insights into understanding the oncogenic functions of *miR-196b-5p* by targeting FAS, and interaction between FAS and STAT3 in the progression of NSCLC. Our study suggested that targeting *miR-196b-5p*/FAS/NFkB/IL6/STAT3 pathway might be effective to treat some NSCLCs.

## Supplementary information

Supplementary Figure 1

Supplementary Figure 2

Supplementary Figure 3

Supplementary Figure 4

Supplementary Figure 5

Supplementary Figure 6

Supplementary Figure 7

Supplementary Tables

Supplementary Figure Legends
