# Supplementary figures and images for: miR-196b-5p-mediated downregulation of FAS promotes NSCLC progression by activating IL6-STAT3 signaling
Source: Cell Death Dis. 2020 Sep 22;11(9):785. doi: 10.1038/s41419-020-02997-7 (PMC7508872; doi:10.1038/s41419-020-02997-7)

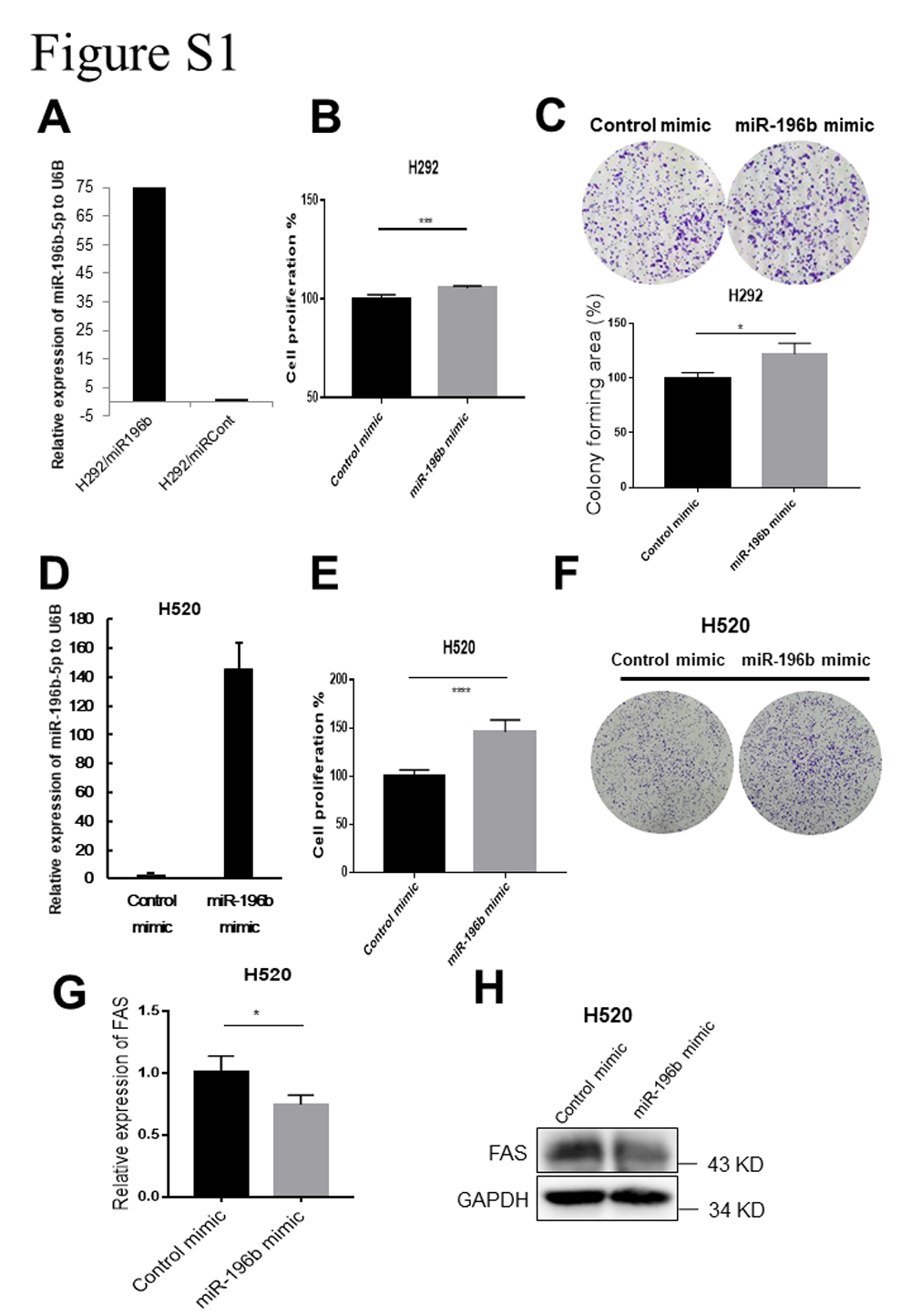

Supplement: Supplementary file 1 — Supplementary Figure 1 [file 41419_2020_2997_MOESM1_ESM.tif]

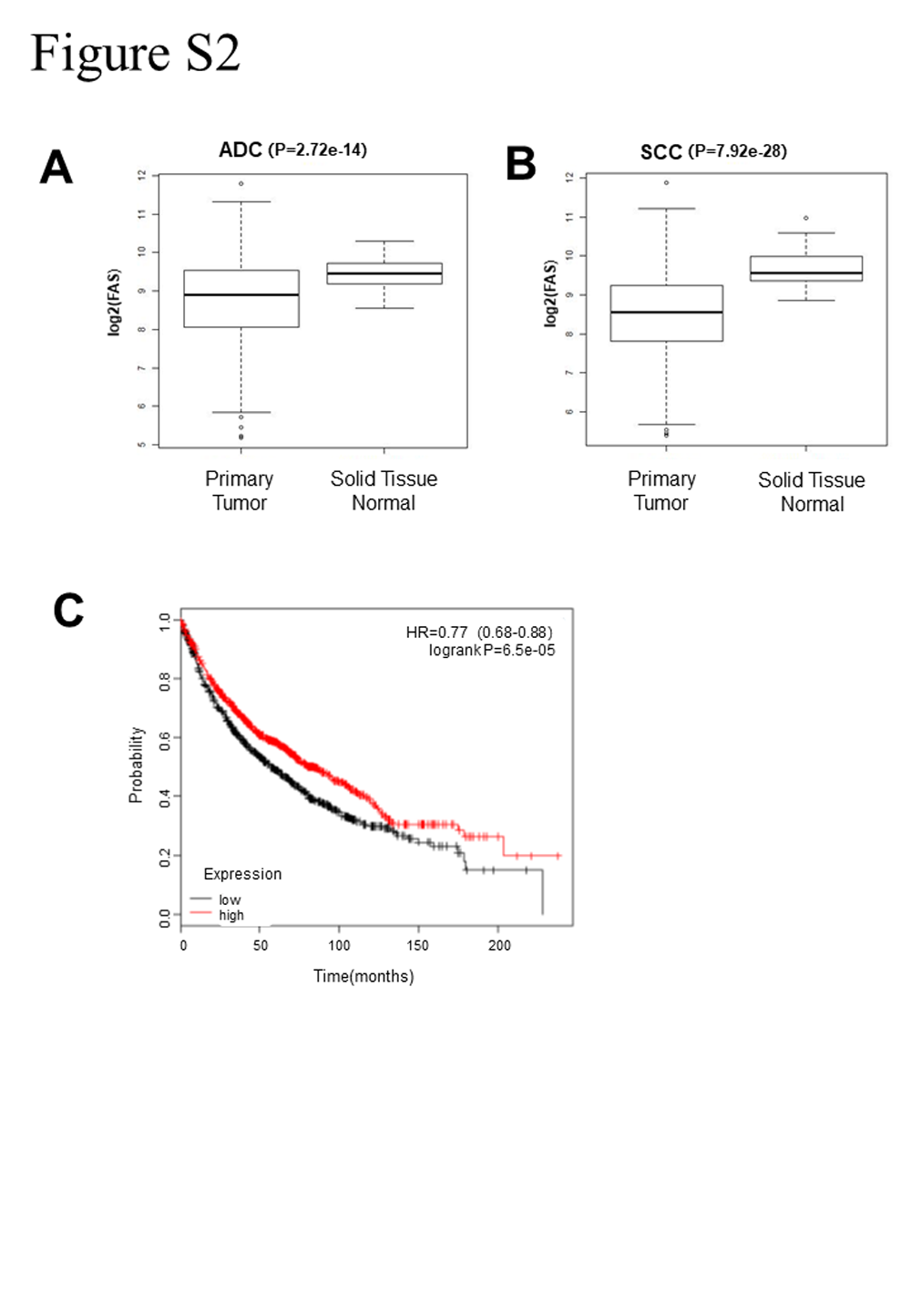

Supplement: Supplementary file 2 — Supplementary Figure 2 [file 41419_2020_2997_MOESM2_ESM.tif]

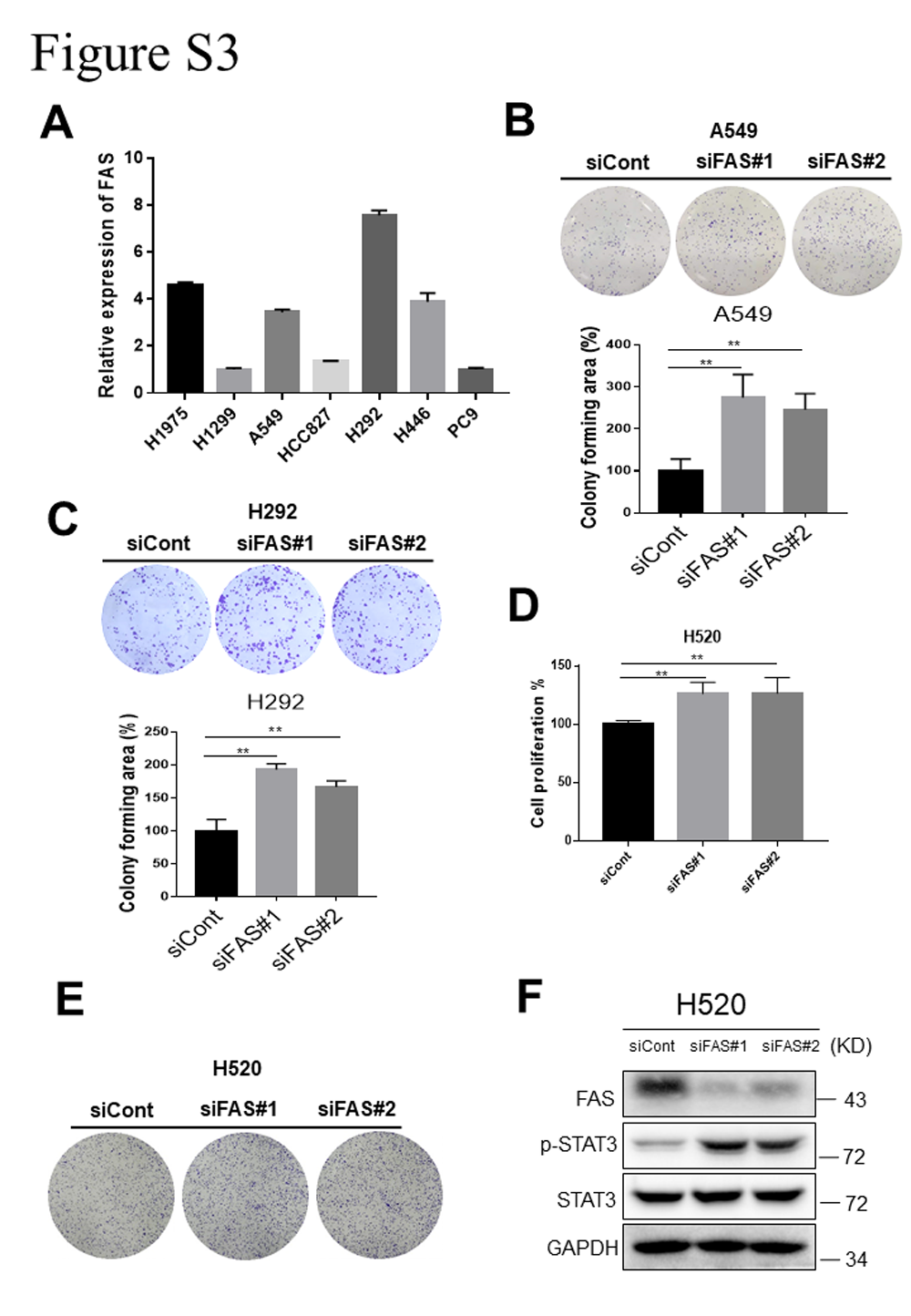

Supplement: Supplementary file 3 — Supplementary Figure 3 [file 41419_2020_2997_MOESM3_ESM.tif]

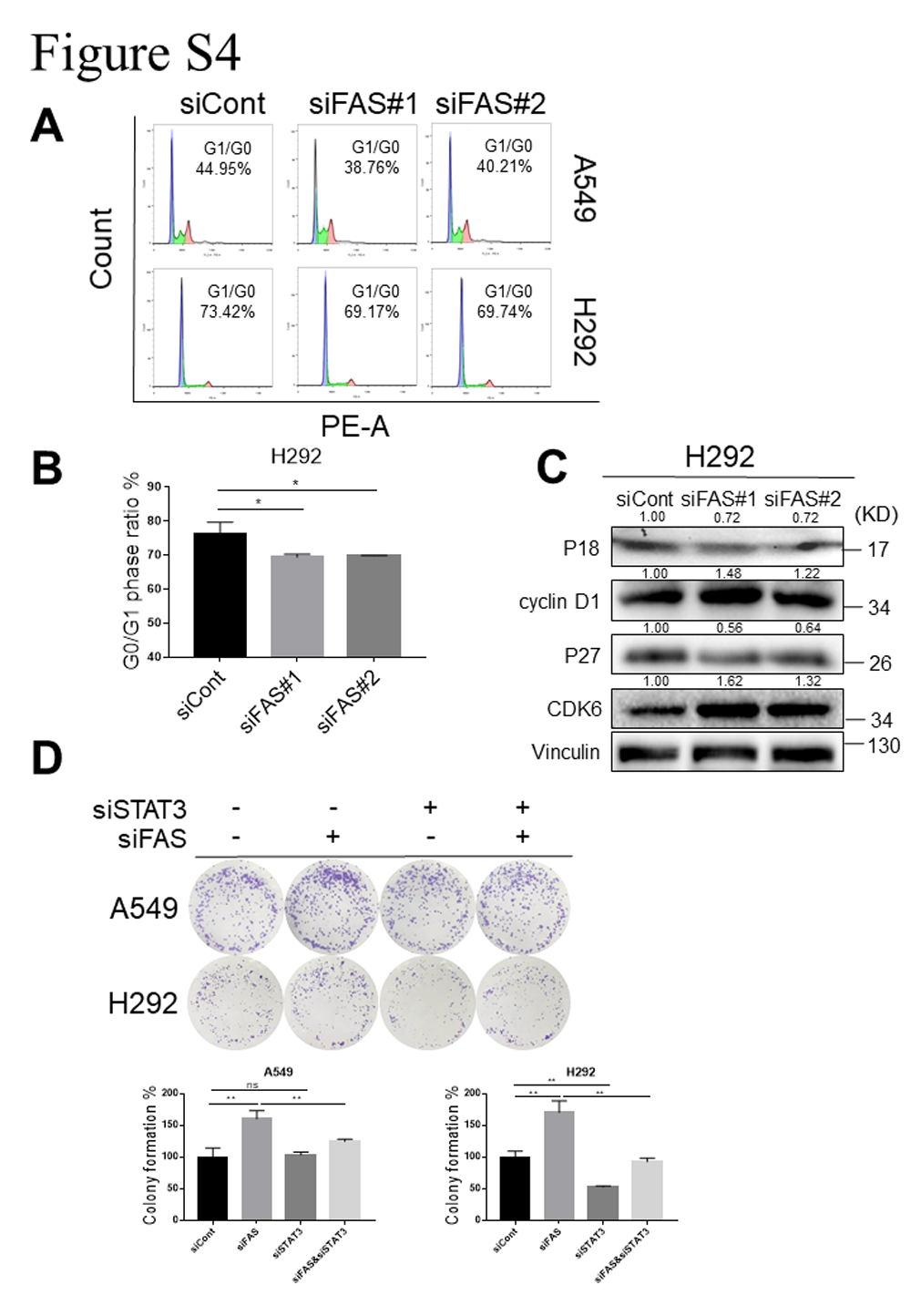

Supplement: Supplementary file 4 — Supplementary Figure 4 [file 41419_2020_2997_MOESM4_ESM.tif]

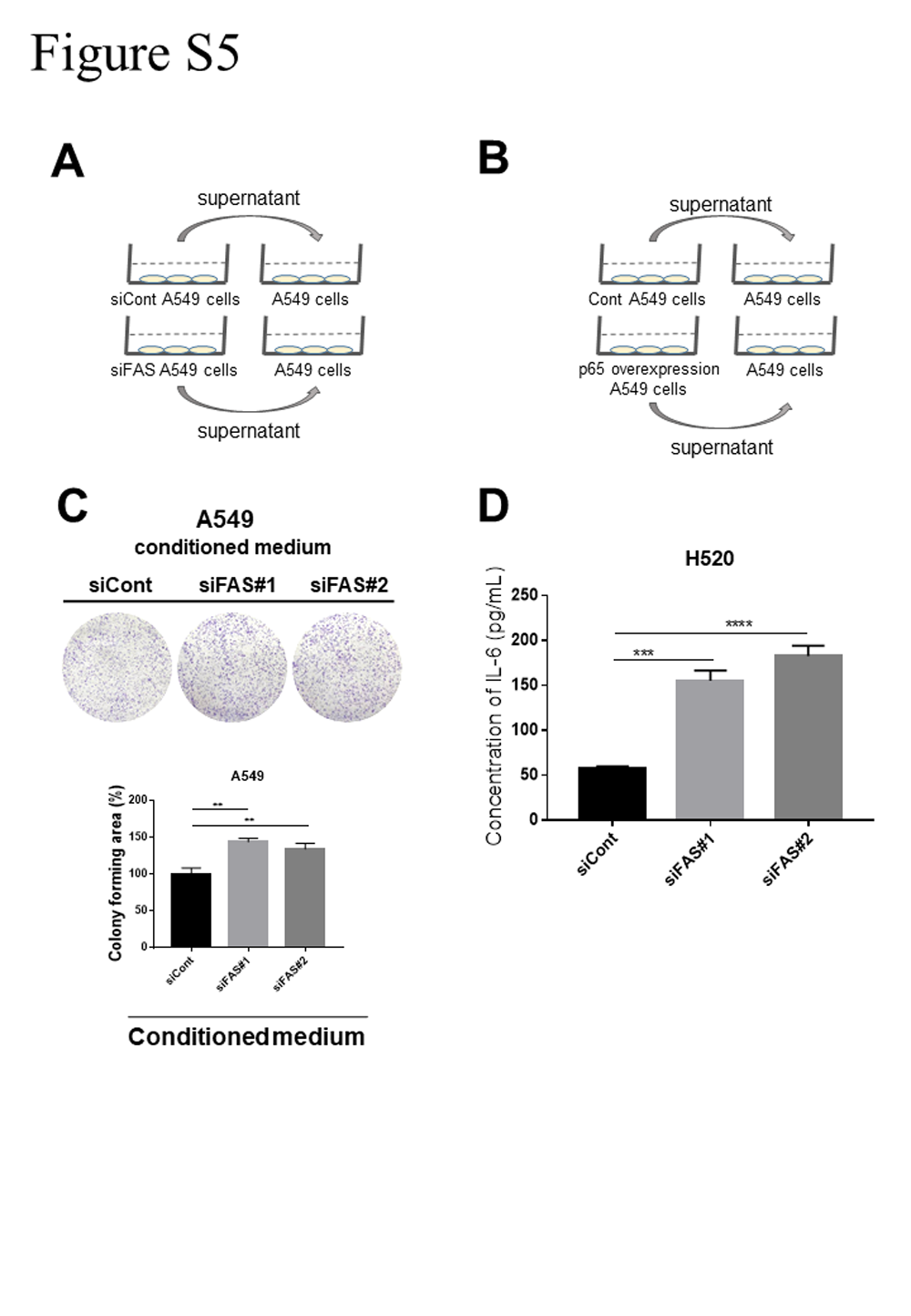

Supplement: Supplementary file 5 — Supplementary Figure 5 [file 41419_2020_2997_MOESM5_ESM.tif]

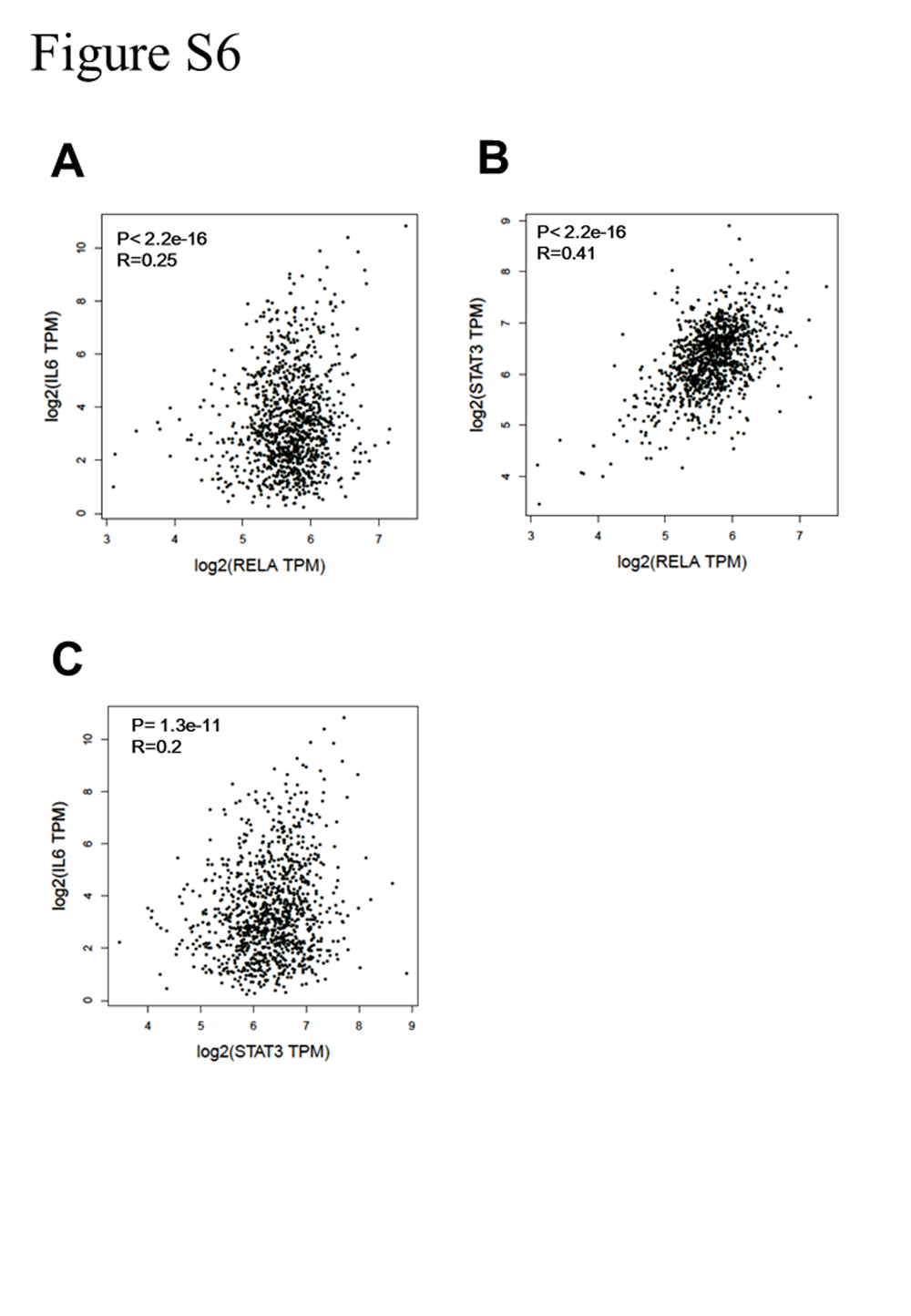

Supplement: Supplementary file 6 — Supplementary Figure 6 [file 41419_2020_2997_MOESM6_ESM.tif]

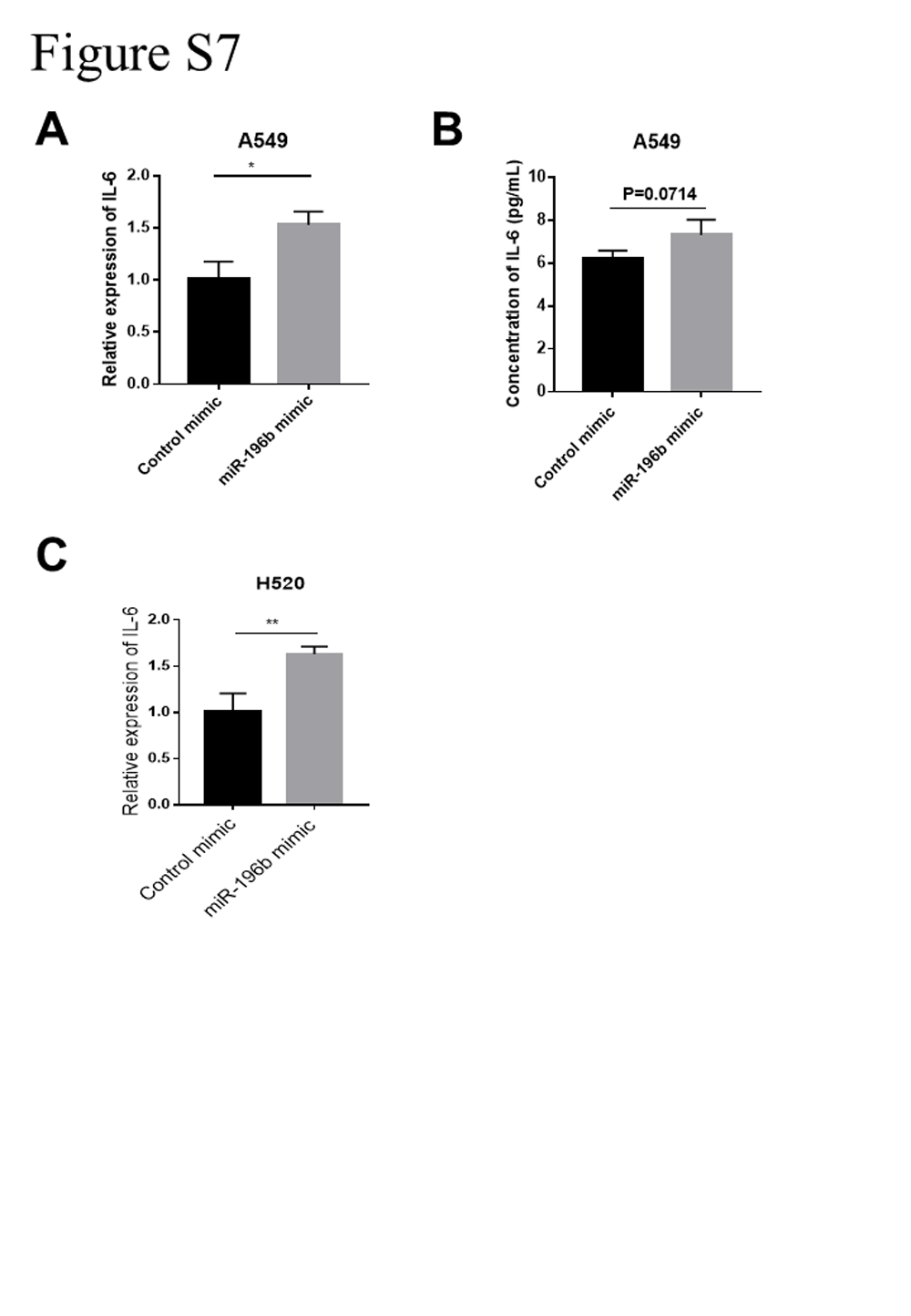

Supplement: Supplementary file 7 — Supplementary Figure 7 [file 41419_2020_2997_MOESM7_ESM.tif]
