# Supplementary material for: miR-196b-5p-mediated downregulation of FAS promotes NSCLC progression by activating IL6-STAT3 signaling
Source: Cell Death Dis. 2020 Sep 22;11(9):785. doi: 10.1038/s41419-020-02997-7 (PMC7508872; doi:10.1038/s41419-020-02997-7)
Supplement: Supplementary file 8 — Supplementary Tables [file 41419_2020_2997_MOESM8_ESM.docx]

**Supplementary Tables**

**Table S1.** Sequences of siRNAs targeting FAS and STAT3.

**Table S2.** qRT-PCR primers for *IL-6*, *FAS* and *GAPDH*.

| Names | Primer sequence (5ʹ-3ʹ) |
| --- | --- |
| IL-6_F | ACTCACCTCTTCAGAACGAATTG |
| IL-6_R | CCATCTTTGGAAGGTTCAGGTTG |
| FAS_F | GGAGTACACAGACAAAGCCCA |
| FAS_R | CTTGGTATTCTGGGTCCGGG |
| GAPDH_F | TCAAGGCTGAGAACGGGAAG |
| GAPDH_R | GACTCCACGACGTACTCAGC |

**Table S3.** Predicted binding sites of miR-196b-5p in 3’-UTR of *FAS* from miRWalk.
