# Supplementary material for: miR-196b-5p-mediated downregulation of FAS promotes NSCLC progression by activating IL6-STAT3 signaling
Source: Cell Death Dis. 2020 Sep 22;11(9):785. doi: 10.1038/s41419-020-02997-7 (PMC7508872; doi:10.1038/s41419-020-02997-7)
Supplement: Supplementary file 9 — Supplementary Figure Legends [file 41419_2020_2997_MOESM9_ESM.docx]

**Supplementary Figure Legends**

**Supp Fig 1** (A) qRT-PCR examines miR-196b-5p expression in miR-196b overexpressing H292 cells and control cells. (B) Cell proliferation assay for H292 after transfection with miR-196b mimic. Results are represented as means ± SD (n≥3). (C) Colony formation assay for H292 cells after transfection with miR-196b mimic. Colony forming areas were measured by Image J software and relative colony forming areas were calculated by comparing with corresponding controls. (D) qRT-PCR examines miR-196b-5p expression in miR-196b overexpressing H520 cells and control cells. (E) Cell proliferation assay for H520 cells after transfection with miR-196b mimic. Results are represented as means ± SD (n≥3). (F) Colony formation assay for H520 cells after transfection with miR-196b mimic. (G) *FAS* expression in H520 cells after overexpressing *miR-196b-5p* was determined by qRT-PCR. Results are represented as means±SD (n=3). (H) Western blot analysis of FAS protein in H520 cells after overexpressing *miR-196b-5p*. *P<0.05, ***P<0.001.

**Supp Fig 2** (A, B) *FAS* expression data from TCGA RNA-seq data in 334 lung ADC patients with 57 matched NATs (A) and 349 lung SSC patients with 51 matched NATs (B). (C) Kaplan-Meier survival analysis using 1882 available NSCLC patients from the Kaplan Meier Plotter.

**Supp Fig 3** (A) qRT-PCR measure *FAS* expression in different lung cancer cell lines. The expression was normalized by *GAPDH*. Results are represented as means±SD (n=3). (B, C) Colony formation assay for A549 and H292 cells after transfection with siFAS for 7 days. Colony forming areas were measured by Image J software and relative colony forming areas were calculated by comparing with corresponding controls. Results are represented as means±SD (n=3). (D) Cell proliferation assay for H520 cells after transfection with siFAS for 4 days. Results are represented as means±SD (n=3). (E) Colony formation assay for H520 cells after transfection with siFAS for 10 days. (F) Western blot analyses for p-STAT3 (Y705) and STAT3 in FAS knockdown H520 cells. **P<0.01.

**Supp Fig 4** (A) Flow cytometry analysis for cell cycle in A549 and H292 after transfection with siFAS for 48h. (B) The percentage of cell cycle G1 phase cells in FAS knockdown H292 lung cancer cells and control cells. Results are represented as means±SD (n=3). (C) Western blot analyses for cell cycle G1 phase related proteins in FAS knockdown H292 lung cancer cells and control cells. The bands were quantified using Image J software and relative values were obtained by normalizing to the value of each corresponding Vinculin. (D) Cell colony formation assay for FAS knockdown A549 and H292 lung cancer cells transfecting with or without STAT3 siRNAs. Colony forming areas were measured by Image J software and relative colony forming areas were calculated by comparing with corresponding controls. Results are represented as means±SD (n=3). *P<0.05, **P<0.01, ns=no significance.

**Supp Fig 5** (A) Schematic illustration of the experimental approach applied for assess the effect of conditioned medium from FAS knockdown cells on the proliferation of lung cancer cells. (B) Schematic illustration of the experimental approach applied for assess the effect of conditioned mediums from p65 overexpressing cells and control cells in lung cancer proliferation. (C) Colony formation assay for A549 cells treated with conditioned medium from FAS knockdown cells and control cells. Colony forming areas were measured by Image J software and relative colony forming areas were calculated by comparing with corresponding controls. Results are represented as means±SD (n=3). (D) ELISA measures secreted IL-6 protein levels in conditioned media from FAS knockdown H520 cells and control cells. Results are represented as means±SD (n=3). *P<0.05, **P<0.01, ns=no significance.

**Supp Fig 6** RELA (p65), IL6 and STAT3 expression data from TCGA RNA-seq data were used to examine the correlation between IL6 and p65 (A), STAT3 and p65 (B) and IL6 and STAT3 (C).

**Supp Fig7** (A) qRT-PCR measure IL-6 expression in miR-196b-5p overexpressing A549 lung cancer cells. The expression was normalized by GAPDH. Results are represented as means ± SD (n=3). (B) ELISA measures secreted IL-6 protein levels in conditioned media from miR-196b-5p overexpressing A549 cells and control cells. Results are represented as means ± SD (n=3). (C) qRT-PCR measure IL-6 expression in miR-196b-5p overexpressing H520 lung cancer cells. The expression was normalized by GAPDH. Results are represented as means ± SD (n=3). *P<0.05, **P<0.01.

**Supplementary Table Legends**

**Table S1.** Sequences of siRNAs used in knockdown analysis.

**Table S2.** Sequences of primers of *IL-6*, *FAS* and *GAPDH* for qRT-PCR.

**Table S3.** Bioinformatical analysis of miR-196b-5p binding sites in 3’-UTR of *FAS.*
